# Non-ergodicity of a globular protein extending beyond its functional timescale†

**DOI:** 10.1039/d2sc03069a

**Published:** 2022-08-04

**Authors:** Jun Li, JingFei Xie, Aljaž Godec, Keith R. Weninger, Cong Liu, Jeremy C. Smith, Liang Hong

**Affiliations:** School of Physics and Astronomy, Shanghai Jiao Tong University Shanghai 200240 China; Interdisciplinary Research Center on Biology and Chemistry, Center for Excellence in Molecular Synthesis, Shanghai Institute of Organic Chemistry, Chinese Academy of Sciences Shanghai 201203 China; University of the Chinese Academy of Sciences Beijing 100049 China; Mathematical BioPhysics Group, Max Planck Institute for Biophysical Chemistry Göttingen 37077 Germany; Department of Physics, North Carolina State University Raleigh NC 27695 USA; UT/ORNL Center for Molecular Biophysics, Oak Ridge National Laboratory Oak Ridge Tennessee 37831 USA; Department of Biochemistry and Cellular and Molecular Biology, University of Tennessee Knoxville Tennessee 37996 USA; Institute of Natural Sciences, Shanghai Jiao Tong University Shanghai 200240 China hongl3liang@sjtu.edu.cn

## Abstract

Internal motions of folded proteins have been assumed to be ergodic, *i.e.*, that the dynamics of a single protein molecule averaged over a very long time resembles that of an ensemble. Here, by performing single-molecule fluorescence resonance energy transfer (smFRET) experiments and molecular dynamics (MD) simulations of a multi-domain globular protein, cytoplasmic protein-tyrosine phosphatase (SHP2), we demonstrate that the functional inter-domain motion is observationally non-ergodic over the time spans 10^−12^ to 10^−7^ s and 10^−1^ to 10^2^ s. The difference between observational non-ergodicity and simple non-convergence is discussed. In comparison, a single-strand DNA of similar size behaves ergodically with an energy landscape resembling a one-dimensional linear chain. The observed non-ergodicity results from the hierarchical connectivity of the high-dimensional energy landscape of the protein molecule. As the characteristic time for the protein to conduct its dephosphorylation function is ∼10 s, our findings suggest that, due to the non-ergodicity, individual, seemingly identical protein molecules can be dynamically and functionally different.

## Abstract 2

Internal motions of folded proteins have been assumed to be ergodic, *i.e.*, that the dynamics of a single protein molecule averaged over a very long time resembles that of an ensemble.

## Graphical abstract

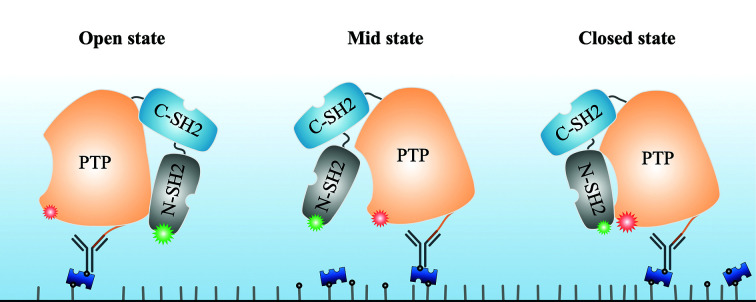

## 1 Introduction

Most functional processes of proteins involve internal motion, often requiring transitions between conformational states.^1–3^ As a globular protein is chemically and structurally highly heterogeneous, this leads to a complex energy landscape over which the protein moves. In turn, a rich variety of motions over the landscape are seen, and these are present over a remarkable time span stretching from femtoseconds up to seconds and beyond. How these motions on different timescales relate to and influence each other, and how the overall characteristics of internal dynamics relate to biological function is of particular interest in biophysics. Also, the intriguing possibility exists that otherwise identical single protein molecules might be physically distinct on timescales approaching their functional times (*e.g.*, enzyme catalytic rates^4,5^). In this regard, a particularly interesting question is whether internal protein dynamics is ergodic, *i.e.*, in the limit of long measurement times, time-averaged observables are equal to its ensemble average. Ideally, non-ergodic means non-converged quantities on all timescales. Clearly, in practice, as a result of temporal limitations on experiments and simulations, all time scales cannot be reached. Therefore, there is no rigorous way of using data obtained on finite timescales to distinguish between non-ergodic and ergodic systems. On finite timescales, true non-ergodicity cannot be distinguished from transient non-convergence. However, even on limited timescales, dynamics can be described using models that are either themselves ergodic or non-ergodic. This distinction is essential because theories of protein function are usually formulated in terms of ensemble averages, and if these are not equivalent to time averages, then they are erroneous. We refer to non-ergodicity on a finite timescale as “observational non-ergodicity”.

Various experiments have demonstrated measurements of the internal dynamics of ensembles of a folded protein under physiological conditions to be non-exponential in time.^3,6,7^ However, this non-exponential (or ‘anomalous’) behavior has been described using ergodic models (such as fractional Brownian motion, where subjective movements of the particle are anti-correlated^6,8–10^) or from non-ergodic models (such as a subdiffusive continuous-time random walk, where the particle is trapped by energy basins that obey a power-law distribution of waiting times without a finite mean^3,11^). Whereas the non-exponential scenario has been found in numerous single-molecule fluorescence experiments and molecular dynamics (MD) simulations,^6,8–10^ non-ergodic interpretations have been relatively unexplored.^3^

The present work focuses on discussing the observational non-ergodicity of a protein observed in the time windows probed by the smFRET experiments (10^−1^ to 10^2^ s) and MD simulation (10^−12^ to 10^−7^ s). Although the systematic experimental exploration of the non-ergodicity of proteins molecule is lacking, its existence is consistent with, and indirectly supported by, experimental observations of static disorder in enzymatic behavior,^12–18^ in which reaction rates of individual enzyme molecules are found to be many-fold different, with the differences sustained for the entire experimental time window (∼hours). Notwithstanding, the vast majority of single-molecule and ensemble experiments have described protein internal motions using ergodic frameworks.^6,19–24^ Whether protein internal motion is non-ergodic on any given timescale remains actively debated among theoretical and computational researchers,^11,25,26^ and its resolution requires thorough experimental tests.

**Fig. 1:**
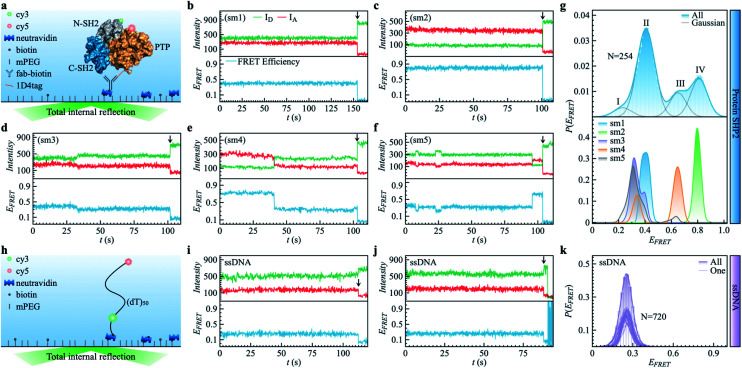
Protein SHP2 and single-stranded DNA (ssDNA) internal dynamics were revealed by smFRET. (a) Schematic diagram of the experimental setup used for the protein single-molecule measurements. The structure of SHP2 contains two Src homology-2 domains (N-SH2, grey; C-SH2, blue) and a PTP domain (gold). Cy3-Cy5 (green and red spheres), a FRET pair of dye molecules, were labeled on residues 87 and 266, *i.e.*, N-SH2 and PTP domain, respectively. The 1D4-tagged protein was immobilized on PEG passivated coverslips through a biotinylated antibody (fab-biotin) and imaged *via* TIRF microscopy. (b–f) Five representative single-molecule (sm) fluorescence trajectories of protein, where the intensities of donor and acceptor dye molecules, *I*_A_ and *I*_D_, are presented in the upper panel while the resulting FRET efficiency, *E*_FRET_ = *I*_A_/(*I*_A_ + *I*_D_), is shown in the bottom panel. The events of photobleaching are indicated by arrows. (g) The overall distribution (top) of FRET efficiency *P*(*E*_FRET_) was obtained from 254 protein smFRET trajectories together with the distributions of *E*_FRET_ for each of the five single-molecule (bottom) trajectories presented in (b–f). The illustrated histogram reveals a shoulder centered at 0.2 (ultra-low FRET, state I) and three major low/mid/high FRET states centered at 0.45 (II), 0.65 (III), 0.8 (IV). The total *E*_FRET_ histogram was fitted with four Gaussian peaks (blue line in top panel). (h) Control experiment of single-stranded DNA (ssDNA) dynamics. ssDNA was labeled with a cy3/5 FRET pair at 50 monomer separations. (i and j) Two experimental ssDNA single-molecule traces. (k) The ensemble-averaged distribution of FRET efficiency (green bars and area) *P*(*E*_FRET_), and FRET histogram of one trajectory (green lines) for the ssDNA. Unlike the SHP2 (g), which can assume several FRET states, the ssDNA shows a single FRET state centering at 0.25 ± 0.05, similar to what was previously reported.^32^

Here, to examine the ergodicity of protein internal dynamics over a range of times, we conduct single-molecule fluorescence resonance energy transfer (smFRET) experiments and all-atom molecular dynamics (MD) simulations on the cytoplasmic protein-tyrosine phosphatase (SHP2). SHP2 is a multi-domain protein (Fig. 1a), participating in multiple cellular signaling processes, including the Ras/MAPK and Hippo/YAP pathways.^27^ As reported recently, SHP2 is prone to liquid–liquid phase separation (LLPS),^2^ in which the proteins coalesce to form condensation droplets different from the surrounding cytoplasmic environment.^2,28^ LLPS of SHP2 has been demonstrated to play a crucial role in regulating and triggering Noonan syndrome (NS),^29^ juvenile myelomonocytic leukemias (JMMLs),^30^ and cancers.^2,27^ Although this protein is used mainly as a model system in the present work to characterize the dynamical heterogeneity in a typical globular protein; there may be some implications for LLPS formation, discussed later.

The present MD simulations and smFRET experiments demonstrate that functional inter-domain motions in the protein show heterogeneity over two wide time windows: from 10^−12^ to 10^−7^ s and 10^−1^ to 10^2^ s. Moreover, as illustrated by control simulations and experiments on a single DNA chain of similar size, which behaves ergodically, we demonstrate how the anomalous dynamics of the protein arises from the characteristic protein energy landscape, which has a much higher dimensionality and unique hierarchical structure. Importantly, biochemical studies have determined that the timescale associated with SHP2 phosphatase activity is tens of seconds.^2^ As the observed non-ergodicity extends beyond this timescale, this could impact the function of this enzyme in its native biochemical signaling network.

## 2 Results

### 2.1. The SHP2 conformational heterogeneity revealed by smFRET

As shown in Fig. 1a, SHP2 contains two Src homology-2 domains (N-SH2, grey; C-SH2, blue), a central PTP catalytic domain (gold), and a C-terminal tail.^2^ The relative motion between the N-SH2 and PTP domains is crucial for its function,^2^ and is characterized here by smFRET experiments on the timescale of 0.1 to 200 s. For these experiments, two selected residues (Q87 and K266), located in the N-SH2 and PTP domains, were labeled with two conjugated fluorescent dye molecules (donor Cy3 and acceptor Cy5, green and red spots in Fig. 1a), and their fluorescence intensities are denoted as *I*_D_ and *I*_A_, respectively. The energy transfer efficiency, defined as *E*_FRET_ = *I*_A_/(*I*_A_ + *I*_D_), is directly related to the inter-dye distance, with a smaller value of *E*_FRET_ corresponding to a longer distance.^31^ Thus, *E*_FRET_ monitors the temporal evolution of the distance between the two labeled residues (additional experimental details are available in the ESI Methods).

We obtained 254 single-molecule FRET trajectories of SHP2, for which the fluorescence intensity of Cy3 and Cy5 are anti-correlated over time, and the trajectories used for analysis were truncated before photobleaching. Five representative single-molecule FRET trajectories are plotted in Fig. 1b–f. As can be seen, over the time window (0–200 seconds) probed, some protein molecules stay in one FRET state (Fig. 1b and c), while others transit between two (Fig. 1d and e) or three (Fig. 1f) distinct states. This behavior indicates that any single protein molecule explores only a portion of the conformational space sampled by the ensemble over the observation time window. To further illustrate this heterogeneity, Fig. 1g compares *P*(*E*_FRET_), the overall histogram of *E*_FRET_, averaged over an ensemble of 254 trajectories (blue, top panel) with those derived from each of the five individual trajectories in Fig. 1b–f. The ensemble-averaged *P*(*E*_FRET_) exhibits three major peaks, at 0.45 (II), 0.65 (III), and 0.8 (IV), with a small shoulder at 0.2 (I), indicating at least four conformational states observed. In contrast, two of the five single trajectories (sm1, sm2) are located in one state, whereas the other three (sm3 to sm5) transition between two or three states in the time window observed.

We note that the differences in the FRET values between the four states are significantly larger than the fluctuations within one state, and are also larger than the fluctuations of fluorescence intensity when the protein is labeled by only one dye molecule (see Fig. S1†). Moreover, for comparison, we also provide the smFRET results of a single-stranded DNA (Fig. 1h–k, experimental details in supplementary information†), denoted as ssDNA, whose radius of gyration (*R*_g_) is ∼3.4 nm, close to that of the SHP2 protein (*R*_g_ ∼ 2.7 nm). The ssDNA presents only one FRET state (Fig. 1k; *E*_FRET_ = 0.25 ± 0.05, for mean ± s.d.), similar to previous reports.^32,33^ All the above comparisons demonstrate that the four observed FRET states of SHP2 result from different conformations of the protein molecule rather than photobleaching, blinking, fluctuation of laser intensity, or any other instrumental or environmental factors.

**Fig. 2:**
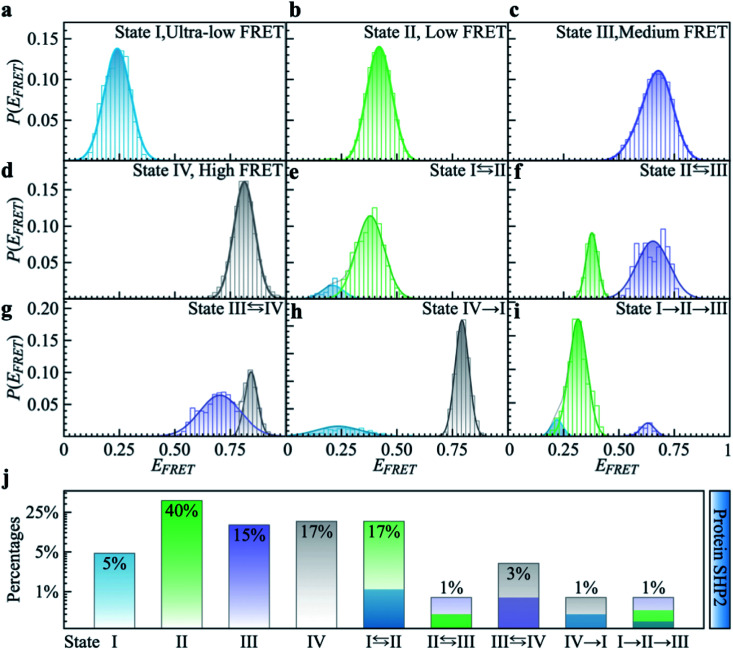
Categorizing the experimental smFRET traces into nine subgroups based on the hidden Markov modeling analysis. Here, 127 FRET traces were chosen from the overall 254 trajectories to ensure each trace lasts at least 100 seconds long, and only the first 100 seconds of the trace were used for analysis. (a–d) *P*(*E*_FRET_) of typical single-molecule example traces for Subgroup I to IV where the protein molecule stays at one state for over 100 seconds. (e–h) Example traces for Subgroup V to VIII where the protein molecule transits between two states over 100 seconds. (i) In the example trace for Subgroup IX the protein molecule transitions between three states over 100 seconds. (j) The relative populations of the nine subgroups summed over 127 traces.

To quantitatively characterize how each single-molecule FRET trajectory explores the four conformational states in the protein, we applied a four-state hidden Markov model (HMM).^34^ Details of the model can be found in the ESI.† The analysis was conducted on 127 trajectories chosen from the overall 254 such that each of them lasted at least 100 s before photobleaching. Only the first 100 s of the chosen trajectories were analyzed to guarantee that the comparison was conducted for the same length of time. The 127 trajectories were categorized into nine subgroups. As can be seen in Fig. 2, subgroups I to IV correspond to the case in which the protein molecule stays in one single state over the entire 100 s (Fig. 2a–d), and subgroups V to VIII correspond to molecules transitioning between two states (Fig. 2e–h) and subgroup IX is the case in which the protein transitions among three states over the 100 s (Fig. 2i). We show the relative populations of these nine subgroups in Fig. 2j. As can be again seen, a significant degree of dynamical or conformational heterogeneity is present among different trajectories. This heterogeneity, *i.e.*, the same species of the molecule exhibiting distinct conformations, and remaining different on the 0.1–200 s time window, is a manifestation of observational non-ergodicity.^35^ The transitions between different FRET states are further analyzed in a transition density plot (Fig. S3†), in which the transitions between states I and II and between states III and IV are most evident.

### 2.2. Observational non-ergodicity in SHP2 measured by MD

**Fig. 3:**
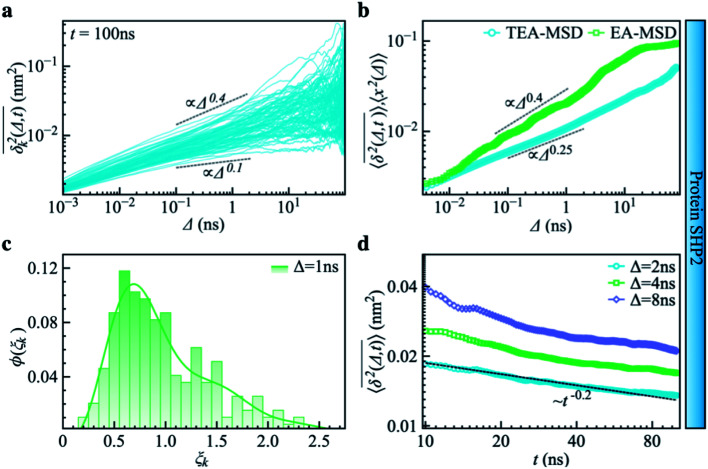
Subdiffusive, non-ergodic, and aging dynamics in SHP2 derived from molecular dynamics (MD) simulations. (a) TA-MSD as a function of lag time *Δ* for each of 100 independent MD trajectories. (b) Comparison of MD-derived ensemble-averaged MSD (EA-MSD, green squares) without time averaging and the time-ensemble-averaged MSD (TEA-MSD, blue circles). The dashed lines indicate asymptotes of power-law fits. These difference between EA-MSD and TEA-MSD directly proves the breaking of ergodicity on the MD time window.^36,37^ The oscillations of EA-MSD are caused by a limited amount of simulation trajectories used for analysis. (c) Scatter distribution, (*ξ*_k_), at *Δ* = 1 ns is skewed with the primary peak located much below 1. (d) TEA-MSD as a function of observation time, *t*, with three fixed lag times *Δ* as indicated (2 ns, 4 ns, and 8 ns). The dashed line in (d) guides the trend of decay.

Complementing the above experiments, we also conducted 100 independent MD simulations of the single protein in an aqueous solution at ambient conditions. Each of these was 100 ns long and started from the same initial structure (details in ESI Methods). To characterize the inter-domain motion of the protein in each single MD trajectory, we calculated the corresponding time-averaged mean-squared atomic displacement (TA-MSD):^36,37^1
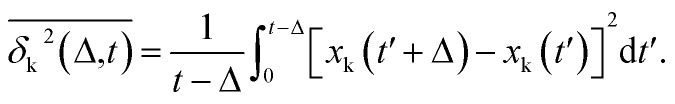
where *x*_k_(*t*′) denotes the distance between two residues Q87 and K266, which defines the inter-domain distance, of the *k*th MD trajectory at time *t*′, *Δ* is the lag-time, and *t* is the time window used for the analysis. As shown in Fig. 3a, 
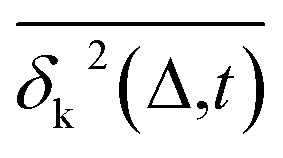
 deviates widely among individual MD trajectories, especially at large *Δ*, indicating considerable dynamical heterogeneity. Fig. 3b compares the time-ensemble-averaged MSD (TEA-MSD, ESI eqn (3)), 
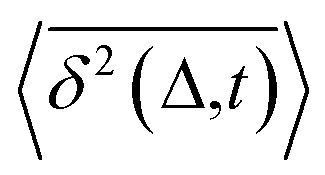
, with that obtained only through ensemble averaging without time averaging (EA-MSD), 〈*δ*^2^(Δ)〉 (ESI eqn (4)). The power-law fits in Fig. 3b suggest the subdiffusive exponents of EA-MSD (*α*_e_) and TEA-MSD (*α*_*t*_) are 0.4 and 0.25, respectively. These two quantities differ considerably from each other, directly confirming the breaking of ergodicity on the time scale probed by the MD (1 ps ∼ 100 ns).^36,37^

Another standard test for ergodicity is the scatter distribution,^36^*ϕ*(*ξ*_k_), where *ξ*_k_ is defined as a dimensionless ergodic-breaking parameter 
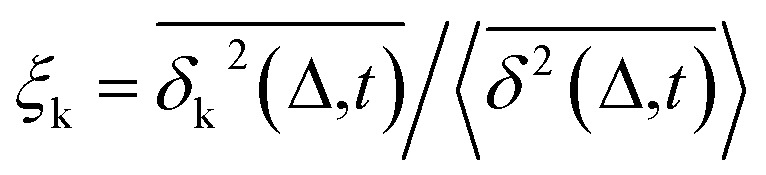
. *ϕ*(*ξ*_k_) gives information on the distribution of TA-MSD among trajectories at a given lag time, *Δ*. For an ergodic or homogeneous system, *ϕ*(*ξ*_k_) will show a narrow peak at *ξ*_k_ = 1, whereas a non-ergodic process will assume a skewed distribution, with the peak located away from 1.^36^ As shown in Fig. 3c, the MD-derived *ϕ*(*ξ*_k_) is rather broad, with the most intense peak located well below 1, indicating the protein molecules in many MD trajectories are highly restrained, displaying flexibilities below the average. Hence, again, the inter-domain motion of the protein is non-ergodic and heterogeneous over the 1 ps ∼ 100 ns time window probed by MD.

Non-ergodic phenomena have been reported in various complex biological systems, such as the diffusion of a nanoparticle in an actin filament network,^38,39^ the lateral movement of protein molecules in the cell membrane,^40,41^ and the transportation of protein granules in the cytoplasm of living cells.^42,43^ Accompanying the non-ergodicity, these systems often show striking aging phenomena in which the effective mobility of the studied particle is reduced upon increasing the observation time,^36,37^ as manifested as a decay of the TEA-MSD over *t* at a given *Δ*. Non-ergodicity is related to the aging properties of the processes involved, that is, the dependence of physical observables on the time span between the initialization of the system and the start of the measurement. Fig. 3d shows the TEA-MSD *vs.* the trajectory length, obtained by truncating the data at an observation time *t* and performing a temporal average (*i.e.*, a moving average). As shown in Fig. 3d, the TEA-MSD decays with *t* as a power-law: TEA-MSD ∼ *t*^−0.2^. Indeed, the internal dynamics of SHP2 ages with the observation time. This aging behavior is often interpreted by the framework of continuous-time random walk (CTRW),^38–43^ and thus why we derive the waiting time distribution in Fig. S7.† We found that the waiting time distributions are broadly distributed as *τ*^−(1+*α*)^ with power-law CTRW exponent *α* = 0.8, indicating CTRW contributes to complicated protein internal dynamics.

The protein's TEA-MSD shows aging and subdiffusion (*α*_*t*_ < 1), which indicates the combination of non-ergodic CTRW and ergodic models as the underlying mechanisms of protein internal dynamics.^36^ The Gaussian distributed step-size function (Fig. S8c†) and anti-persistency velocity correlation function (Fig. S8d†) of protein inter-domain distance *x*(*t*), suggest ergodic FBM subordinated to the CTRW. If we assume a free diffusion is adopting a mixed origin of CTRW and FBM. This implies the relation of *α*_e_ = *αβ* and *α*_*t*_ = 1 − *α* + *αβ*, where *α* is the power-law exponent of the waiting time, and *β* is twice of the Hurst exponent. In the present work, as *α* = 0.8 is smaller than 1 (see Fig. 3d in the main text); and *α*_*t*_ should be larger than *α*_e_, contradicting the results in Fig. 3b. We note that *α*_e_ > *α*_*t*_ was also found in various biological systems.^44–47^ These works often attribute this finding to the confinement effect.^44,45^ Confinement is unambiguously present in the present work as the studied object is the distance between the two domains of the protein SHP2, which is structurally constrained. Moreover, as revealed in ref. 11, the underlying energy landscape is self-similar and fractal. All these could lead to the observation of *α*_e_ > *α*_*t*_.

### 2.3. The difference between non-convergence and observational non-ergodicity

**Fig. 4:**
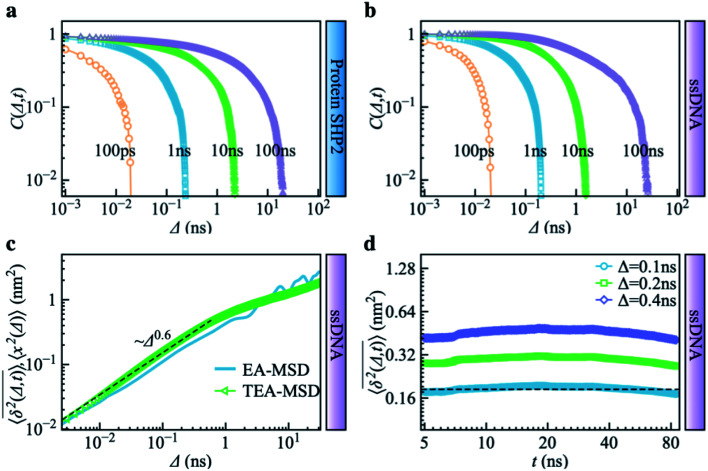
Control simulation on a single-stranded DNA (ssDNA). The normalized autocorrelation functions of distance fluctuation were obtained from molecular dynamics simulations for different trajectory lengths (*i.e.*, 100 ps, 1 ns, 10 ns, 100 ns). Both (a) protein SHP2 and (b) ssDNA show non-converged dynamics. (c) MD-derived EA-MSD (blue) *vs.* TEA-MSD (green) for the ssDNA. (d) TEA-MSD of ssDNA as a function of observation time, *t*, with three fixed lag times *Δ*.

Combining Fig. 1–3, one can conclude that the inter-domain motions of SHP2 are heterogeneous over wide time ranges: 10^−12^–10^−7^ s for the MD and 0.1–200 seconds for the smFRET. Given the broad distribution of relaxation timescales for internal protein motions, one might wonder whether the observed non-ergodic dynamics in the protein results from non-convergence, *i.e.*, that the observed time window is shorter than the longest relaxation time in the system.^48^ To explore this question, we carried out MD simulations on the single-stranded DNA (ssDNA), whose smFRET experimental results were displayed in Fig. 1h–k. Here, the normalized autocorrelation functions (ESI eqn (6)) were calculated from the simulation trajectories to measure the convergence of the systems. As seen in Fig. 4a and b, both SHP2 and ssDNA exhibit non-converged dynamics, *i.e.*, the autocorrelation function decays progressively slower when prolonging the time window for analysis, with no convergence from 1 ps to 100 ns. Moreover, the characteristic times of protein autocorrelation functions show a linear dependence on the measurement time (Fig. S9†). Besides, as seen in Fig. S10,† the distributions of the characteristic distance in both the protein and ssDNA vary significantly with the observation time, further confirming the non-convergence of the dynamics in the two systems on the time scale explored (1 ps to 100 ns). However, for ssDNA, its TEA-MSD and EA-MSD almost overlap (Fig. 4c), revealing no appreciable non-ergodicity. Such ergodic behavior in ssDNA derived from MD is consistent with the smFRET experimental results on it (see Fig. 1h–k), where all individual ssDNA molecules stay in one FRET state over ∼100 s. Moreover, further analysis of the MD trajectories shows no significant aging in ssDNA (Fig. 4d). These results demonstrate that although both the protein and ssDNA albeit exhibit non-converged MD dynamics (Fig. 4a and b), the absence of non-ergodicity in the ssDNA (Fig. 4c and b) is qualitatively different from the non-ergodic behavior of the protein (Fig. 3). The experimental and simulation results suggest that the dynamics of ssDNA is ergodic. This is consistent with the simulation findings on a short peptide, chignolin, which has only 10 amino acids without strongly-fixed secondary or tertial structures and also exhibits ergodic dynamics up to tens of microseconds.^49^ One might deduce that the complex structure of the protein studied here, which has a well-defined secondary and tertiary structure, is the key to exhibiting non-ergodic behavior.

Non-converged dynamics can result from two phenomena. One of these is long memory in dynamics beyond observational time. For example, fractional Brownian motion with an infinitely long memory will never converge but will itself be ergodic.^50^ The other phenomenon is the existence of too many distinct conformational states for a single protein molecule to sample over the observation time, *i.e.*, observational non-ergodicity.^11,51^ Hence, by comparing the dynamical behavior of the ssDNA, one can unambiguously conclude that non-convergence alone cannot cause the non-ergodicity observed in SHP2 protein.

### 2.4. The energy landscape of protein SHP2 and a single-stranded DNA

**Fig. 5:**
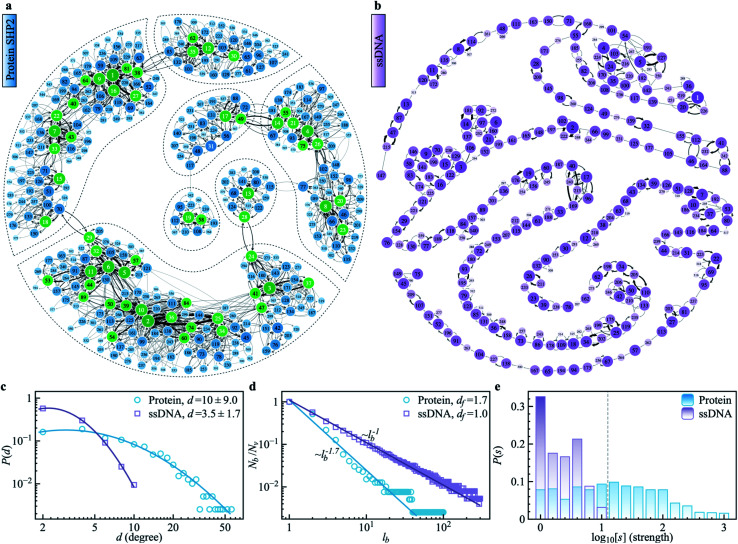
Conformational cluster transition network (CCTN) of the protein SHP2 and ssDNA. (a) The network was produced using a 1 μs MD simulation trajectory with each snapshot saved at every 10 ps. Each vertex represents one conformational state, corresponding to a group of protein conformations with a similar structure as defined by the cutoff value of RMSD. Here the cutoff is chosen as 1.6 Å, to ensure the number of vertices in each CCTN is comparable with each other and falls in the range of 200–500. The network has 396 vertices and 2120 edges. In the CCTN, conformational states with higher transition probability are arranged closer to each other. The darkness of the color indicates its occurrence rate, calculated by counting the total number of snapshots belonging to the cluster. The vertices mark with an integer in terms of the rank of occurrence probability. The directed edges denote a transition between two conformational states observed in MD and are weighted by the associated transition probability. The networks representing the energy landscape were produced using the Python module graph-tool. The green vertices correspond to the most visited nodes (strength, *s* > 100) in the protein network. Such heavily visited nodes are absent in ssDNA. The dashed lines highlight the hub regions, where the internal nodes are densely connected with each other, but only a few paths are connected to the outside. (b) The CCTN of the single-stranded DNA (ssDNA) was derived using a similar method as above, using a 1 μs simulation with snapshots saved at every 10 ps. It shows a string-like feature with 319 nodes and 564 transitions, and the RMSD cutoff is 4.0 Å. (c) The degree distributions *P*(*d*) were derived from the protein SHP2 (blue) and ssDNA (purple) transition network in (a) and (b), respectively. The blue and purple lines represent log-normal fits (ESI eqn (8)). The mean values and standard deviations of connecting degrees are displayed in the legend. (d) We applied a box covering algorithm to the CCTN to derive the fractal dimension of the transition networks in (a) and (b). The number of boxes (*N*_b_) required to cover the CCTN normalized by the total number of nodes (*N*_v_) in the network, is plotted as a function of the box's length, *l*_b_. The power-law fit (blue) suggests the underlying protein energy landscape is a self-similar fractal with a dimension ∼1.7. While the number of boxes (*N*_b_) shows a linear relationship with box length (*l*_b_) for the ssDNA network in (b), indicating the energy landscape of ssDNA is relatively flat with the one-dimensional geometry of CCTN rather than fractal. (e) The histograms of strengths *P*(*s*), *i.e.*, the distribution of frequency of each node being visited observed in MD, were obtained from the protein SHP2 (blue) and ssDNA (purple) transition network in (a) and (b), respectively.

To explore the protein phase space in detail, following the procedure of ref. 11, we constructed a conformational cluster transition network (CCTN) based on a single MD trajectory,^11,52,53^ describing conformational transitions of the protein molecule (Fig. 5a and b). Briefly, we assigned all protein conformations sampled to different conformational clusters based on their structural similarity as quantified by the root mean square deviation (RMSD) (more details in ref. 54 and the caption to Fig. 5). In the CCTN, a node corresponds to one conformational cluster, the population of which is given by the number of MD frames in it. A node with a darker color represents a cluster with a higher population. Edges with an arrow denote observed transitions between two conformational states, and the thickness of the arrow represents the transition probability. Thus, the CCTN coarse grains a continuous MD trajectory into discretized transitions between conformational states on the energy landscape.^11,52,53^

An example of a CCTN obtained from the MD trajectory of the protein is presented in Fig. 5a. The network is highly complex and inhomogeneous, forming loosely connected hubs (see the regions enclosed by the dashed lines), where the inter-hub connections are rather limited, but the nodes inside the hub are densely connected with each other. We also performed the same network analysis from the MD trajectory of ssDNA (Fig. 5b). Compared to the protein, the CCTN of the ssDNA is much simpler, with most nodes having only two neighboring nodes and connected linearly without forming many hubs.

To quantitatively examine the connections in the networks of the two systems, we analyzed the degree distribution, *P*(*d*), *i.e.*, the probability distribution of the number of connections per node.^55^ As shown in Fig. 5c, *P*(*d*) of the protein is much broader than that of the ssDNA, where the width of the distribution is 9.0 in the protein, about 5 times wider than that in the ssDNA (s.d. = 1.7). And the majority of nodes in the protein CCTN have more than 10 connecting neighbors, far more than that in the ssDNA (mean = 3.5). Moreover, one can examine the topological structure of the networks. Here, we applied a box covering method (see details in ESI†) to estimate the fractal dimension.^55,56^ The fractal dimension determined for the protein network is 1.7 (Fig. 5d, blue), consistent with an earlier study on another protein, phosphoglycerate kinase, for which the value was found to be 2.4.^11^ In contrast, the fractal dimension of the ssDNA is about 1.0, indicating it resembles a one-dimensional linear network (Fig. 5d, purple). We also compared the node strength (*s*), *i.e.*, the frequency of visiting each node in the network.^57^ As shown in Fig. 5e, the CCTN of the protein has many heavily visited nodes (*s* > 100), which are the center nodes of the hubs (highlighted in green in Fig. 5a). In contrast, such heavily visited nodes are absent for ssDNA. This results from the hierarchical structure of the energy landscape of the protein in which the protein frequently visits the nodes inside any given hub but takes a long time to escape out as relatively few transition paths connect to external hubs. As a result, long-lived metastable conformations of the protein result (see Fig. 1 and 2).

The above comparative analysis reveals that the SHP2 protein has a much more complex energy landscape than the ssDNA, with a higher dimensionality and a much more hierarchical structure, and the conformational states have many more connecting neighbors. We note there exist many local structures and constraints (*e.g.*, α-helix and β-sheets, which are stabilized by intrachain hydrogen bonding, disulfide linkages, ionic bonding, *etc.*) inside the structure of the protein, which will limit the protein conformational changes. All these features lead to the protein molecule having many different pathways to transit between any two distant states, and also lead to it staying in single metastable states, the hub centers, for long times. This network structure leads to heterogeneous dynamics among individual protein molecules observed over a long period of time, *i.e.*, observational non-ergodicity.

Finally, we note that the timescales explored by MD simulations (10^−12^ to 10^−7^ s) and by single-molecule FRET experiments (0.1 to 200 s) differ by six orders of magnitude. However, as shown in Fig. S11,† both the topological structure and the degree distribution of the CCTN of the protein are scale-free, *i.e.*, independent of whether the simulation is 100 ns or 1 μs long. This scale-free and self-similarity character of the energy landscape was shown earlier for several different proteins over many decades in time.^11^ Hence, we attribute the non-ergodic dynamics in the protein to its characteristic high-dimensional, hierarchical, self-similar complex energy landscape. We note that an unambiguous confirmation of such non-ergodicity observed in simulation can extend to the experimental time window that can not be accessed by all-atom MD simulations. It might be able to be examined by the coarse-grained simulation, *e.g.*, ref. 58, which is beyond the present work and could be carried out in the future.

## 3 Discussion and conclusion

The analysis of dynamics over a finite time window does not permit a determination of the ergodicity of the system on infinite timescales.^48^ Therefore, it is only meaningful to discuss non-ergodicity over a certain observational time window, *i.e.*, observational non-ergodicity, and this is what is examined in the present work. Observational non-ergodicity has been documented on the time window of 0.01–100 seconds in various biological phenomena, including the transport of protein molecules or nanoparticles through complex macroscopic biological systems, such as cell membranes, living cells, and actin filaments.^38–43^ These systems are large enough (>1 μm) and have structures that are complex and heterogeneous enough to produce complex, non-ergodic dynamics. Single-molecule force-clamp spectroscopy has demonstrated non-ergodicity to occur when unfolding a protein molecule at the time window of 0.01–10 s.^18,59^ However, unfolding or folding corresponds to a dramatic perturbation of the biomolecule, far away from its folded globular functional state. Here, we demonstrate that observational non-ergodic dynamics is also present in the internal motions of a small globular protein in its physiological folded state over a timescale longer than the characteristic time for the protein to perform its dephosphorylation function.^2^ Comparison with the simulation and experimental results of a control system, a single-strand DNA of similar size, illustrates that non-convergence alone can not cause the observed non-ergodic dynamics in the protein. Rather, non-ergodicity results from the high-dimensional, hierarchical connectivity in the energy landscape of the protein.

Dynamical heterogeneity on functional timescales, due to relaxation processes existing on these timescales or longer, will theoretically lead to functional differences. The observed dynamical heterogeneity in the protein is thus likely to lead to the population splitting of individual enzyme molecules with theoretically different catalytic rates.^4,5^ This is consistent with the experimental observation of “static disorder” of enzymatic rates among individual enzyme molecules, in which the catalytic rates of individual enzyme molecules can be many-fold different, with the differences sustained for hours.^12–16,18^ Moreover, one can see from Fig. 1g that the protein is trapped in very different conformational states for tens or hundreds of seconds. Such long-lived diverse conformational states could trap the SHP2 protein molecules in different conformations for sufficiently long times to diffusively find a partner with complementary shape and electrostatic interactions, leading to association and, in turn, triggering the liquid–liquid phase separation (LLPS) for which this particular protein is known.^2^

A final, intriguing question arises as to whether observational non-ergodicity among individual protein molecules will disappear when the observation time extends beyond hundreds of seconds probed here. For a single protein in an aqueous solution, at some point in time, the folding: unfolding equilibrium will be well sampled, and if one ignores degrading chemical reactions, one would then expect ergodicity to be reached. However, this question cannot be addressed in this work. Further, an experimental work on another multi-domain protein,^3^ Hsp90, using plasmon rulers has revealed extremely long-lived (∼12 hours) open and closed configurations. The extent of non-ergodicity in internal motions of proteins of different structures and functions and the biological implications of this will be a topic for future research.

## 4 Methods

We used prism-type total internal reflection fluorescence (TIRF) microscopy for measurement as described previously.^2,60^ Data were recorded with a time resolution of 100 ms for all cases (SHP2, Donor only, and ssDNA). The coverslip was coated with polyethylene glycol and biotinylated PEG (mPEG-SVA and Biotin-PEG-SVA, molar ratio 97 : 3). Then, fluorescently labeled and 1D4 tagged proteins were immobilized *via* a biotinylated antibody (Fab-biotin, anti-1D4tag) attached through neutravidin to the passivated quartz slides (Fig. 1a). This immobilization scheme has been reported for other proteins in studies of their dynamics and functions.^61^ The biotinylated ssDNA was directly immobilized through neutravidin to the coverslips (Fig. 1h). The smFRET experiments were performed at room temperature of 25 °C. The protein sample was prepared in a working buffer (500 mM NaCl, 50 mM HEPES, 2 mM TECP, 5% glycerol at PH 7.5). The experiment was incubated for 10 min before image acquisition started. Subsequent single-molecule videos were measured in imaging solution (75 mM NaCl, 75 mM KCl, 50 mM HEPES, 0.5 mM TCEP at pH 7.5) for protein, and T50 buffer for ssDNA. An enzymatic deoxygenation system (0.625% wt/vol glucose, 0.8 mg ml^−1^ glucose oxidase, 0.03 mg ml^−1^ catalase, 3 mM Trolox) was added into the buffer to alleviate the fluorescent photobleaching and blinking.^2^

## 5 Measurements

Methods of single-molecule protein and single-stranded DNA sample preparation, Cy3/Cy5 labeling, smFRET data analysis, and molecular dynamics simulations, and related theoretical analysis were described in ESI.†

## Data availability

All the data are shown in the ESI.†

## Author contributions

L. H. conceived the project.; J. L., J. F. X., performed research; J. L. analyzed data; and J. L., J. F. X., A. G., K. R. W., C. L., J. C. S., and L. H. wrote the paper. J. L., A. G., J. C. S., and L. H. discussed the results and provided input on the manuscript.

## Conflicts of interest

The authors declare no competing interests.

## Supplementary Material

SC-013-D2SC03069A-s001
