# Supplementary material for: Non-ergodicity of a globular protein extending beyond its functional timescale
Source: Chem Sci. 2022 Aug 4;13(33):9668–77. doi: 10.1039/d2sc03069a (PMC9400594; doi:10.1039/d2sc03069a)
Supplement: SC-013-D2SC03069A-s001 [file SC-013-D2SC03069A-s001.pdf]

Electronic Supplementary Material (ESI) for Chemical Science.
This journal is © The Royal Society of Chemistry 2022

Supplementary Information for

Non-ergodicity of a globular protein extending beyond its functional timescale

Jun Li, JingFei Xie, Aljaž Godec, Keith R. Weninger, Cong Liu, Jeremy C. Smith, Liang Hong*

***Corresponding Author:** Liang Hong; **Email:** hongl3liang@sjtu.edu.cn

This PDF file includes:

Supplementary Text

1. Single-molecule FRET measurement
2. All-atom molecular dynamics (MD) simulations
3. Protein inter-domain distance $x(t)$ distributions
4. Definition of different types of mean-squared displacement
5. Different subdiffusive models and their (non-)ergodic or aging behavior
6. The simulation and experimental waiting time distributions of protein conformational states
7. FBM noise subordinated to CTRW
8. Autocorrelation functions and characteristic relaxation times of SHP2 protein
9. Log-normal distributed degree distribution
10. Box covering algorithm for the estimation of the fractal dimension

Supplementary Figures

Fig. S1. Control smFRET experiment of the donor only labeled SHP2

Fig. S2. Representative single-molecule FRET trajectories of protein SHP2

Fig. S3. Transition density plot for SHP2 dynamics

Fig. S4. The structures of globular protein SHP2 and single-stranded DNA

Fig. S5. Protein SHP2 inter-domain distance $x(t)$ distributions

Fig. S6. Subdiffive internal dynamics of protein SHP2 and ssDNA

Fig. S7. The waiting time distributions $P(\tau)$ of protein SHP2

Fig. S8. The mixed origin of CTRW and FBM in protein SHP2 internal dynamics

Fig. S9. Autocorrelation functions and characteristic times (τ_c) of SHP2 protein

Fig. S10. The time-dependent distance probability density functions

Fig. S11. The properties of transition network at different trajectory lengths

Supplementary Table

Table S1. The sequence of single-stranded DNA studied by smFRET

Supplementary References

Supplementary Text

1. Single-molecule FRET measurement

The single-molecule fluorescence resonance energy transfer (smFRET) data of SHP2 were taken in Dr. Cong Liu's group.[1,2] Detailed sample preparation and data analysis are described below. Through an engineered tRNA/aminoacyl-tRNA synthetase system, an E76A mutant of SHP2 with two Azido-p-Phe incorporated at Q87/K266 was produced. The expressed protein was concentrated to 5 μM in labeling buffer (500 mM NaCl, 50 mM HEPES, 0.1mM TECP, 5% glycerol at PH 7.5). Then, a mixture of Sulfo-alkyne-Cy3 and Sulfo-alkyne-Cy5 (lumiprobe) 1:1.2 (cy3/cy5) was added into the protein solution with the ratio of 40:1 (dyes/protein). Adding 1.5 mM Tris and 300 μM $CuSO_4$ into the solution, the FRET pair of dye molecules (Cy3 and Cy5) were conjugated to Azido-SHP2 87/266 via a click reaction.[3] 4 mM sodium ascorbate was added into the reaction mixture and followed by 30 ℃ for 40 min. The fluorescently labeled protein was isolated by desalting column (Sephadex-G25, GE healthcare) using the isolation buffer (50 mM HEPES, 500 mM NaCl, 0.5 mM TCEP, and 5% glycerol, pH 7.5) to remove the free dyes and reduce hydrophobic binding of the fluorophores to SHP2. The dual-labeling efficiency was measured as 39.9 % for SHP2. More detailed protocols can be found in Ref. 1,2,4. The preparation of donor-only labeled SHP2 follows the same procedure described above, except the labeling reaction included only one dye molecule (Cy3).

The control experiments used donor-only labeled protein, and single-stranded DNA (ssDNA, dT_{50}) oligonucleotides (sequences in Supplementary Table 1) purchased from Sangon Biotech Co., Ltd (Shanghai, China). We used the T50 buffer (50mM NaCl, 10mM Tris-Cl at PH 8.0) for the smFRET experiment on the ssDNA experiment. All single-molecule studies were repeated at least three times, and no significant difference was shown among these experiments. The results for the two controls are presented in Fig. 1h-k and Fig. S1.

We used prism-type total internal reflection fluorescence (TIRF) microscopy for measurement as described previously.[1,2] Data were recorded with a time resolution of 100 ms for all cases (SHP2, Donor only, and ssDNA). The coverslip was coated with polyethylene glycol and biotinylated PEG (mPEG-SVA and Biotin-PEG-SVA, molar ratio 97:3). Then, fluorescently labeled and 1D4 tagged proteins were immobilized via a biotinylated antibody (Fab-biotin, anti-1D4tag) attached through neutravidin to the passivated quartz slides (see Fig. 1a in the main text). This immobilization scheme has been reported for other proteins in studies of their dynamics and functions.[5] The biotinylated ssDNA was directly immobilized through neutravidin to the coverslips. The smFRET experiments were performed at room temperature of 25 ℃. The protein sample was prepared in a working buffer (500 mM NaCl, 50 mM HEPES, 2mM TECP, 5% glycerol at PH 7.5). The experiment was incubated for 10 min before image acquisition started. Subsequent single-molecule videos were measured in imaging solution (75 mM NaCl, 75 mM KCl, 50 mM HEPES, 0.5 mM TCEP at pH 7.5) for protein, and T50 buffer for ssDNA. An enzymatic deoxygenation system (0.625% wt/vol glucose, 0.8 mg/ml glucose oxidase, 0.03 mg/ml catalase, 3 mM Trolox) was added into the buffer to alleviate the fluorescent photobleaching and blinking.[1]

Preprocessing of all single-molecule videos was performed with the iSMS package using the program's default settings.[6] Cy3 and Cy5 spots were detected using the intensity threshold of 100, and a 2-D Gaussian function fitting was used to identify the fluorescent spots on the EMCCD. The background signal, which was the average intensity of all pixels with a 2-pixel distance to the

fluorescent spot, was subtracted. We removed the blinking traces (due to the oxygen scavenger system, few blinking traces were observed) or the trajectories that showed a sudden multilevel drop from the analysis. Next, only the single-molecule trajectories with the anti-correlated fluorescence intensities between Cy3 and Cy5 confirming energy transfer between the two dyes were selected for further analysis. Additional examples of single-molecule traces of SHP2 are provided in Fig. S2. The FRET efficiency, E_{FRET}, was calculated via the following equation:[7]

$$E_{FRET} = I_A / \left(I_A + I_D \right) = \frac{1}{1 + \left(R / R_0 \right)^6} . \quad (1)$$

Here, I_D and I_A represent the raw data of donor and acceptor fluorescence intensities, respectively, where R is the inter-dye distance, and R_0 is the characteristic distance at which $E_{FRET} = 0.5$, $R_0 = 5$ nm for a Cy3/Cy5 pair.[7] As E_{FRET} is closely related to the inter-dyes distance, the temporal evolution of E_{FRET} can be used to monitor the distance fluctuation between the two labeled residues and thus between the two domains N-SH2 and PTP in SHP2 (see Fig. 1a in the main text). Further analysis of E_{FRET} state sequences was performed using four-state hidden Markov models.[8]

2. All-atom molecular dynamics (MD) simulations

The crystal structure of SHP2 with the E76A mutation can be found in the PDB data bank file 5XZR[4]. The molecular mass of this multi-domain phosphatase is about 64.24 kD with ~530 residues, and the corresponding R_g is about 2.7 nm (Fig. S4a). All SHP2 simulations were carried out using GROMACS (Version 2016.3) with the CHARMM27 force field for this enzyme.[9,10] The system was solvated in a rectangular water box (edge lengths $8.5 \times 9.5 \times 10.5$ nm^3) with periodic boundary conditions (PBC), leading to a total system size of about 83,000 atoms with a single SHP2 protein molecule and ~25,000 water molecules. 77 Na^+ and Cl^- ions were added to mimic the experimental conditions. All simulations were carried out using the TIP3P water model [11] in the NVT ensemble using a Nośe-Hoover thermostat at 300 K.[12] The pressure coupling was performed using the Parrinello-Rahman algorithm with a coupling time of $\tau = 2$ ps.[13] Van der Waals interactions (VdW) were truncated at 1.0 nm, with the LJ potential switched to zero gradually from 1.0 nm to 1.2 nm. The short-range electrostatic interactions within the cut-off distance of $r_c = 10$ Å were treated as Coulombic.[14,15] All bonds involving hydrogen atoms were constrained with the LINCS algorithm to allow a time step of 2 fs.[16] The system was first energy minimized using steepest descent steps with a maximum force of 10.0 kJ·mol^{-1}·nm^{-1} and a maximum of 5×10^6 steps, then equilibrated in the NVT ensemble at 300 K for 10 ns, and then in the NPT ensemble at $p = 1$ bar for 10 ns. Then using the final structure of the protein molecule is obtained in the NPT equilibration as the starting structure. We performed 100 independent MD simulations with each being 100 ns long. One microsecond-long simulation was performed to produce the conformational transition cluster network (Fig. 5a).

The single-stranded DNA (ssDNA) studied here is polyadenylic acid with 40 monomers of thymine (poly dT_{40}, Fig. S4b), and its radius of gyration (R_g) is about 3.2 nm. The starting configuration for the MD study is generated by the x3dna module (http://web.x3dna.org). The GROMACS (version 2016.3) tools package,[17] was used in conjunction with the AMBER99SB-ILDN 96 force field to model ssDNA dynamics.[18] ssDNA was solvated in a periodic box of dimensions $15.5 \times 15.5 \times 15.6$ nm^3 at 300 K temperature and 1 bar pressure in an aqueous solution. This contained approximately 168,000 TIP3P models water molecules with the appropriate amount of Na^+ counterions to neutralize the negative

phosphate charges,[11] and the total system size was about 504,000 atoms. Periodic boundary conditions (PBC) were applied in all dimensions with long-range electrostatic interactions characterized by Particle Mesh Ewald (PME) method.[14,15] The pressure/temperature coupling was performed using the Parrinello-Rahman algorithm,[13] and Nosé-Hoover methods,[19] respectively. MD simulation was carried out using the leap-frog algorithm for integrating Newton's equation of motion for 100 ns at constant temperature (300 K) and pressure (1 bar). Van der Waals interaction was truncated at 1.2 nm, with the LJ potential switched to zero gradually at 1.0 nm.[15] The short-range electrostatic interactions within the cut-off distance of r_c = 12 Å were treated as Coulombic.[14,15] Following that, 10 independent 100 ns long and one 1μs long simulations were produced with a time step of 2 fs integration, and the coordinates of the system coordinates were saved at every 1 ps. The energy minimization, NVT, and NPT preparation follow the same procedure as protein. One 1 μs simulation were used to generate the transition network (Fig. 5b). We used the end-to-end distance of ssDNA to characterize its internal dynamics (Fig. 4b-d).

3. The experimental and simulation protein inter-domain distance *x*(*t*) distributions

We plot the simulation and experimental inter-domain distance distributions $P(x)$ in Fig. S5. The mean values and standard deviations of measured $x(t)$ are displayed in the insert. $P(x)$ of the experiment (Fig. S5b) is much broader than that of the simulation data (Fig. S5a), where the width of the distribution is 0.68 in the experiment, about three times that in the simulation (s.d. = 0.19). And the average distance in the protein SHP2 obtained experimentally is 4.91 nm, much larger than the simulation's $x(t)$ (mean = 2.49). We note that the time scale of the smFRET experiment is much longer (~ 100 s) than that accessed by the MD (~ 1 μs), and thus the conformational space explored by the experiment is expected to be much broader. Moreover, the distance measured in MD is the neat distance between the two residues of the protein, while the $x(t)$ in the experiment is between the two labeled dye molecules, whose size is ~ 1 nm. The latter will significantly enlarge the absolute distance and its flexibility measured by experiment compared to MD.

4. Definition of different types of mean-squared displacement

Given that the distance between the two selected residues (Q87 and K266) in the protein varies with time as x, one can calculate the corresponding time-averaged mean-square displacement (TA-MSD) for a given kth trajectory as:[20]

$$\overline{\delta_k^2(\Delta,t)} = \frac{1}{t-\Delta}\int_0^{t-\Delta}\left[x_k(t'+\Delta) - x_k(t')\right]^2 dt' . \tag{2}$$

where Δ is the lag-time, and overline "-" denotes the time average over the observation time, t. If more than one trajectory is available, one can derive the time-ensemble-averaged mean-squared displacement (TEA-MSD) as:[20]

$$\left\langle \overline{\delta^2(\Delta,t)} \right\rangle = \frac{1}{N}\sum_{k=1}^{N}\overline{\delta_k^2(\Delta,t)} . \tag{3}$$

where N is the number of the simulation trajectories, and the bracket ⟨ ⟩ corresponds to the ensemble average. Note that we use overlines throughout the paper to represent time averages and brackets ⟨ ⟩ to indicate averages over an ensemble of trajectories.

Moreover, one can also calculate the ensemble-averaged mean-square displacement (EA-MSD) without time averaging as:

$$\left\langle \delta^2(\Delta)\right\rangle = \left\langle \left[x_k(\Delta) - x_k(0)\right]^2 \right\rangle . \tag{4}$$

Fig. 3b in the main text compares TEA-MSD with the EA-MSD for the inter-domain motions in SHP2.

When we examined the TEA-MSD at a shorter timescale, as shown in Fig. S6, the crossover behavior emerged for these two systems of protein SHP2 and ssDNA. The high power-law exponent, $\alpha_t > 1$, at a short time scale is often attributed to the ballistic motion of the atoms.

5. Different subdiffusive models and their (non-)ergodic or aging behavior

The subdiffusive motion can be attributed to either a non-ergodic scenario,[20,21] which means that the time-averaged dynamics of a single-molecule are not equivalent to the ensemble-averaged motions, or the ergodic scenario.[20,21] The most popular non-ergodic model is the continuous-time random walk (CTRW),[22] where the particle is trapped by energy basins that obey a power-law distribution of waiting times without a finite mean. Fractional Brownian motion (FBM),[23] where subjective movements of the particle are anti-correlated, and random walk on a fractal structure (RWF),[24] are two popular models for the ergodic scenario.[20,21] CTRW can be separated from FBM and RWF by examining the ergodicity and aging phenomenon.[21] Aging means the effective mobility of the studied particle moves slower when observed longer[21], which is a typical feature for CTRW, but not for the FBM or RWF. The difference between FBM and RWF can be separated by characterizing the Gaussianity of the displacement, as it is the feature of FBM.[21]

6. The simulation and experimental waiting-time distributions of protein conformational states

We found our observed aging behavior has been reported in various biological components.[25-30] These studies have been commonly modeled with the continuous-time random walk (CTRW) framework to elucidate non-ergodic parts, sometimes mixed with ergodic models. Thus, we have evaluated the simulation and experimental waiting-time distributions, and displayed these results in Fig. S7.

For MD simulation, since all conformational states in the conformational cluster transition network (CCTN) can be identified, one can quickly determine the trapping time τ of each state, i.e., the duration of the protein molecule staying at one state before jumping out. Fig. S7a presents the distribution of waiting times, $P(\tau)$, for the protein molecule to reside in each state. As can be seen, $P(\tau)$ presents a fractional power-law as $\tau^{-(1+\alpha)}$ with an exponent of $\alpha = 0.8$.

For the single-molecule experiment, we use the hidden Markov modeling (HMM) method [8] to define the observed four FRET states. One representative single-molecule trajectory is plotted in Fig. S7b, where the gray line is the HMM-defined states, and τ_1 and τ_2 are the waiting times. The obtained waiting time distribution of all FRET states is plotted in Fig. S7c. Due to limited transition events occurring at the measured time window (0.1 - 200 s), this distribution displays a significant degree of scattering. Nevertheless, a power-law fit gives that $\tau^{-(1+\alpha)}$ with $\alpha = 0.8$.

Overall, the distributions of the thus-obtained $P(\tau)$, for simulation and experimental states (Fig. S7) exhibit a fractional power-law scaling as $\tau^{-(1+\alpha)}$, where α is ~ 0.8. This analysis indicates that the waiting time obeys a broad distribution without a finite mean. However, we note that, as the time window analyzed is not broad enough, one can not decisively conclude whether the distribution obeys a fractional power-law or multi exponentials.

7. FBM noise subordinated to CTRW

The protein's TEA-MSD shows aging and subdiffusion (α_t < 1), which indicates the combination of non-ergodic CTRW and ergodic models.

To dissect this mixed origin of protein internal dynamics, we further analyzed the detailed aging behavior of inter-domain distance $x(t)$. In Fig. S8a, the TEA-MSD is plotted against observational time t at 13 different lag times Δ (color gradient). As can be seen, the aging phenomenon diminishes as one reduces the lag time (Δ). We plot the aging exponent (α) as a function of lag time, whose absolute value is reduced from ~0.4 to 0 when reducing the Δ from 10 ns to 1 ps. This observation cannot be elucidated by a simple confined CTRW,[6] but is consistent with the theory of noisy CTRW.[7]

To further reveal which kind of noise was coupled with CTRW, we analyzed the Gaussianity of the step size. The probability of step size exhibited a Gaussian shape for the SHP2 protein, evidenced by the best Gaussian fits (Fig. S8c; blacked line), suggesting Gaussian noise rooted in SHP2 dynamics.

We also calculated the velocity autocorrelation function (VCF) to further dissect the noise type. The VCF is defined as follows: [1,8]

$$C_v^\delta(\tau) = \frac{1}{\delta^2}\left\langle \left(x(\tau+\delta) - x(\tau)\right)\cdot\left(x(\delta) - x(0)\right)\right\rangle . \tag{5}$$

Where the velocity $v(\tau) = \delta^{-1}[x(\tau+\delta) - x(\tau)]$ at time τ is characterized during time periods δ. We plotted the normalized VCF of the SHP2 over different δ, using a color gradient for clarity (Fig. S8d). The VCF appeared to have anti-correlated behaviors, and this shape of VCF is strongly associated with FBM.[31] Hence, anti-correlated step motions together with Gaussian step size suggested that an ergodic FBM noise coupled with CTRW in protein dynamics.

8. Autocorrelation function and characteristic relaxation time

For any physical parameter, e.g., $x(t')$, whose value fluctuates over time, the normalized autocorrelation function (ACF) obtained at different observation times is given by $C(\Delta,t)$[36].

$$C'(\Delta,t) = \frac{1}{(t-\Delta)}\int_0^{t-\Delta} \delta x(t')\cdot\delta x(t'+\Delta)dt' . \tag{6}$$

$$C(\Delta,t) = C'(\Delta,t)/C(0,t). \tag{7}$$

where Δ is the lag time, t is the length of the time window used for analysis, $\delta x(t')$ represents the $x(t')$ fluctuation away from its time average. To improve the statistics of the simulation results, the autocorrelation function is averaged over 100 single-molecule simulation trajectories. As seen in Fig. 4a, b, $C(\Delta,t)$ does not converge and slows down upon an increase of t. Hence, the internal dynamics of

two molecules (protein SHP2 and single-stranded DNA, Fig. 4a, b) are not converging, slowing down upon the increase of observation time, t.

We calculated the experimental and simulation normalized autocorrelation function (ACF) $C(\Delta;t)$ of the inter-domain distance in the protein and presented the results in Fig. S9a and Fig. S9b. One also can estimate the characteristic correlation time (τ_c) as a function of the measurement time (t) (see Fig. S9c). As can be seen, ACF shifts towards longer lag times with increasing observation time t. Accordingly, τ_c increases in a linear relationship with the trajectory lengths, i.e., $\tau_c(t) \sim t$, showing no sign of convergence (Fig. S9c) over fourteen orders of magnitudes in time.

9. Log-normal distributed degree distribution

We calculate the degree distribution, $P(d)$, to examine the topological feature of the transition networks. $P(d)$ is defined as the probability of a vertex linked to d direct neighbors. We plot the degree distribution $P(d)$ in Fig. 5c, which a log-normal distribution can well represent:

$$P(d)=\frac{1}{\sqrt{2\pi}\sigma d}exp\left\{-\frac{\left[\ln(d)-\mu\right]^2}{2\sigma^2}\right\}. \tag{8}$$

where σ and μ are the standard deviation and mean of the distribution, respectively. Notably, all $P(d)$ almost overlap on different trajectory lengths or RMSD cutoff, indicating topological self-similarity.[37] Log-normal degree distribution is characteristic of random multiplicative processes,[38] and is easily observed in scale-free networks and self-similar systems.[39]

10. Box covering algorithm for the estimation of the fractal dimension

We extract information from the energy landscape that is represented by the conformational cluster transition network (CCTN). Particularly, we are interested in the fractal scaling of the CCTN graph. Therefore, a version box covering algorithm for the complex networks, the so-called compact-box-burning (cbb) algorithm,[37,40] was applied to the CCTN to derive the fractal dimension. Here, the "distance" r_f of a graph is defined as the shortest number of edges connecting two vertices, and considering the graph average "mass" M is proportional to the number of nodes (N_v). If the geometry of the network is fractal, the average mass, $\langle M \rangle$ covered by different box length l_b, should follow the power-law relationship:

$$\langle M \rangle \sim \frac{N_b(l_b)}{N_v} \sim l_b^{-d_f}. \tag{9}$$

For a given box length l_b, an integer, N_b is the least number of boxes that can fully cover this network, N_v is the total number of vertices for normalization, and power-law exponent d_f represents the fractal dimension. The examination of the fractal is presented in Fig. S11.

Supplementary Figures

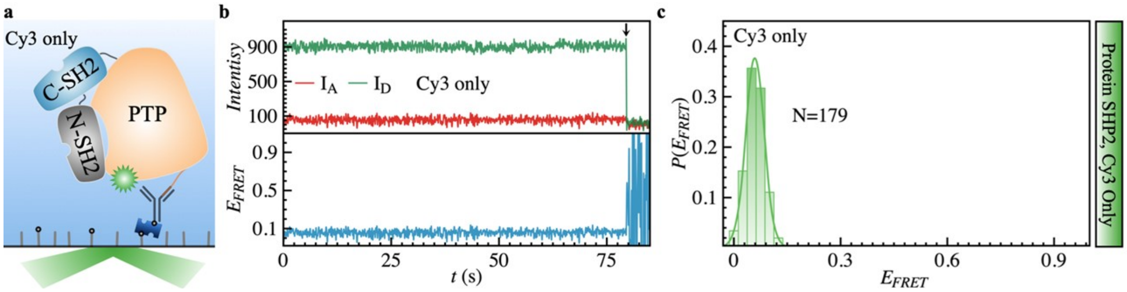

Fig. S1 | Control experiment. single-molecule FRET experiment of the donor only labeled protein. **(a)** Schematic diagram of the SHP2 protein labeled by a donor (cy3, green) only and its experimental setup. (**b)** Example intensity trace of the donor-only sample (green, I_D), where the rather low intensity of the acceptor (red, I_A) results from the leakage of the donor fluorescence into the detecting channel of the acceptor. The intensity of I_A and I_D are positively correlated and disappear at the same time (arrow). **(c)** The resulting $P(E_{FRET})$ is narrowly distributed and centered below 0.1.

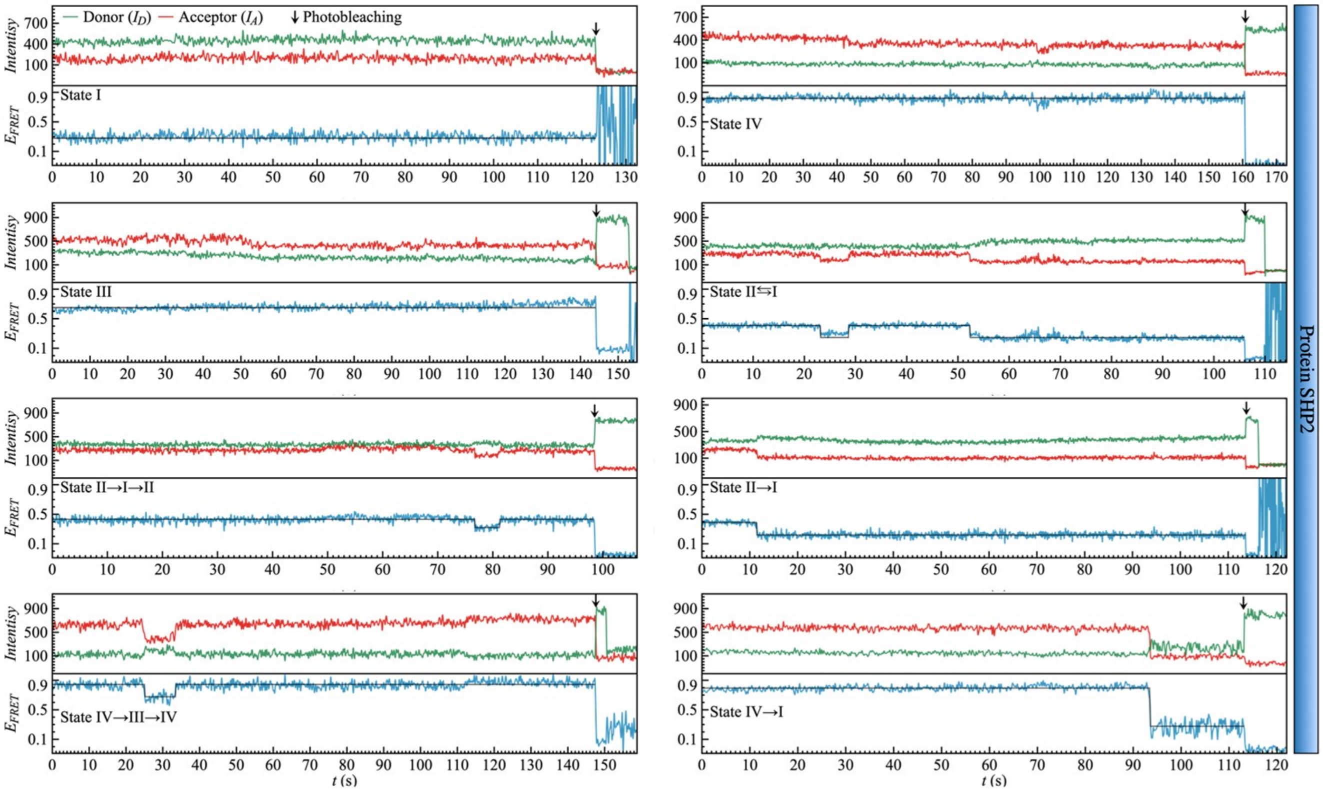

Fig. S2 | Representative single-molecule FRET trajectories of SHP2. The energy transfer of two dye molecules' fluorescence intensities is present. Here, green and red curves are associated with Cy3 (Donor, I_D) and Cy5 (Acceptor, I_A) intensity. The blue line is the corresponding FRET efficiency, which is directly calculated by the intensity ratio, $E_{FRET} = I_A/(I_A+I_D)$. The grey-colored region highlights the data after photobleaching which is removed before analysis. The coarse-grained FRET-state sequences (solid black lines) are derived using hidden Markov modeling (HMM), i.e., a widely used standard method to model the smFRET trajectories by discretized states [8].

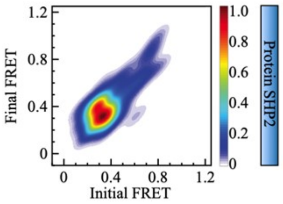

Fig. S3 | Transition density plot for the SHP2 protein derived from the HMM on the corresponding smFRET results. A total of 120 transitions were identified, and the color bar denotes the counts of transitions.

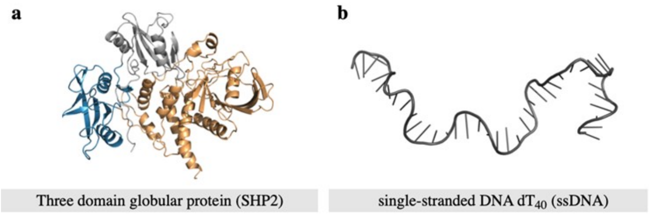

Fig. S4 | The structures of globular protein SHP2 and single-stranded DNA. (a). The crystal structure of protein tyrosine phosphatase SHP2, which contains two Src homology-2 domains (N-SH2, gray; C-SH2, blue) and a protein-tyrosine phosphatase (PTP, gold) domain, PDB code: 5XZR. **(b).** A single chain of 40 monomers of single-stranded DNA (ssDNA) poly dT_{40} from the simulation snapshots.

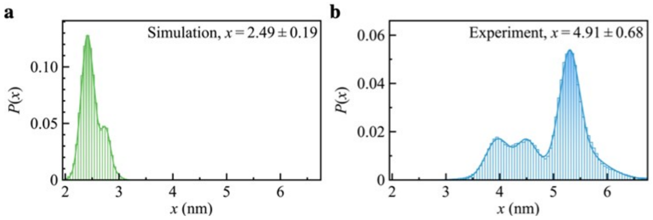

Fig. S5 | Protein SHP2 inter-domain distance *x*(*t*) distributions. The simulation (a) and experimental (b) histograms of $x(t)$. The mean and standard deviation of the inter-domain distance distributions are shown in the inset.

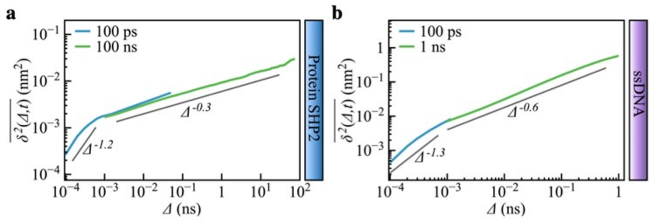

Fig. S6 | Subdiffive internal dynamics of protein SHP2 and ssDNA. (**a**) TEA-MSD averaged over four observational times, t = 100 ps, and t = 100 ns. Gray reference lines indicating power laws with different exponents are plotted as a visual guide. (**b**) The same way visualized TEA-MSD vs. Δ for ssDNA dynamics.

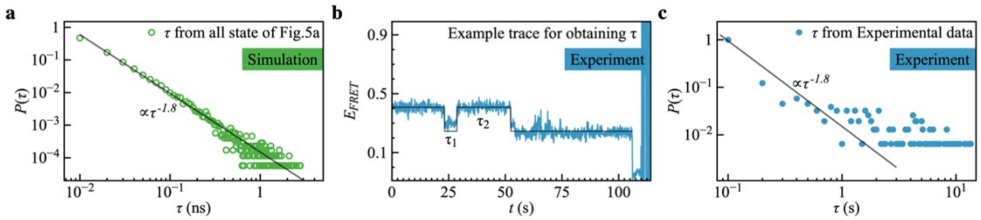

Fig. S7 | The waiting time distributions *P*(τ) of protein SHP2. (a) Distribution of waiting times for the protein molecule to stay in all states of Fig. 5a (main text). Here, we define the trapping time as the time interval when the protein conformation is located in one conformational cluster. **(b)** Example trajectory shows the method to analyze waiting times in the smFRET experiment. The initial and ending parts were not considered for the analysis of dwelling times. **(c)** The waiting time distribution from all FRET states. The data is scattering, due to the limited amount of state transition occurring in trajectory over the observed time window. The blacked lines in (a, c) are power-fits for guidance.

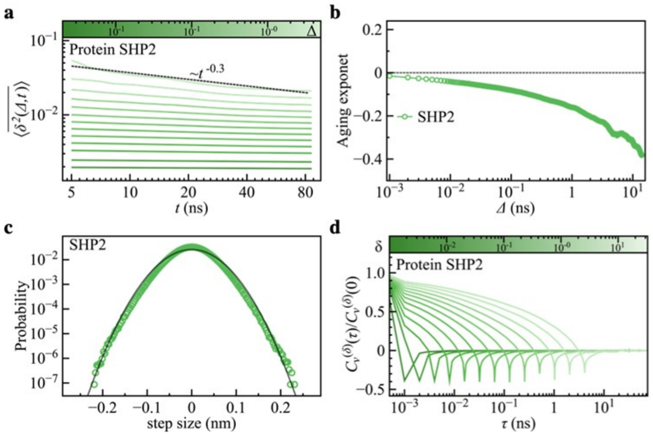

Fig. S8 | The mixed origin of CTRW and FBM in protein SHP2 internal dynamics. (a) The aging TEA-MSD are plotted against observation time, *t* (i.e., the time used in the moving average), with 13 different fixed lag times *Δ* (1 ps - 5 ns) as indicated (colored bar). The power-law fit (dashed line) represents the scaling of TEA-MSD ~ t^{α}, with slope α = - 0.3 at Δ = 5 ns. This exponent indicates aging behavior at the observed timescale. **(b)** The curve of the aging exponent shows that α is dependent on lag time *Δ*, consistent with the theory of noisy CTRW model [22]. **(c)** The step-size distribution of protein SHP2 was obtained from all simulated trajectories with a time step of 1 ps. The overlap between the data (marker) and the fit (line) demonstrates that the noise is Gaussian distributed. **(d)** Normalized velocity correlation function (VCF) for the protein SHP2. The plot consists of 13 different time steps of δ (1 ps - 16 ns) over which the velocity was calculated. δ is visualized as a color gradient for clarity. The observed anti-persistency motions further reveal the existence of fractional Gaussian noise in the protein fluctuation.

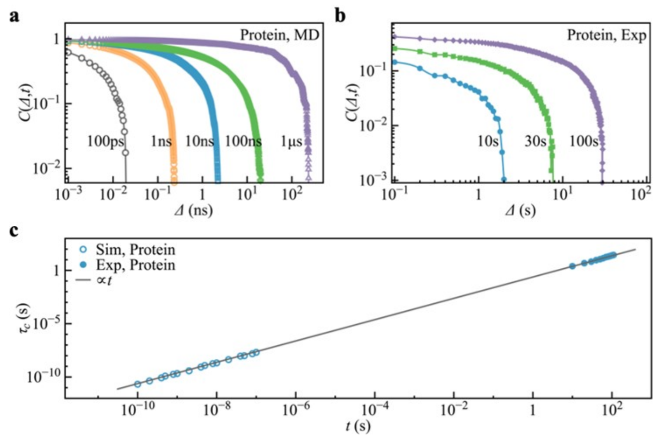

Fig. S9 | Autocorrelation functions and characteristic times (τ_c) of SHP2 protein internal dynamics. (a-b) The normalized autocorrelation functions (ACF) of inter-domain distance fluctuation were obtained from protein simulation and experimental data for different observational times (i.e.,100 ps, 1 ns, 10 ns, 100 ns, 1 µs, 10 s, 30 s, 100 s). **(c)** The characteristic relaxation times τ_c of the protein inter-domain distance fluctuation, are plotted against the observation time, t. Here, τ_c obtained from MD simulations and experiments is defined as the time at which the normalized ACF decays to 0.1. A reference line for the linear scaling $\tau_c(t) \sim t$ is plotted as a visual guide.

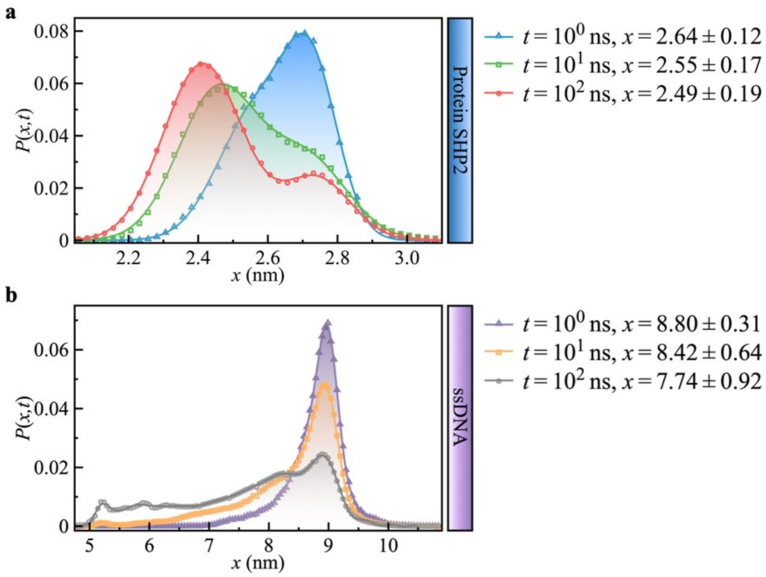

Fig. S10 | The dependence of the distribution of the characteristic distance in (a) SHP2 and (b) ssDNA on the observation time, *t*, derived from MD. Here, the characteristic distance in the protein is defined as the inter-domain distance, while it is the end-to-end distance in the ssDNA. The mean and standard deviation of the distributions are shown in the legend. As can be seen, the distribution, its mean, and standard deviations vary significantly with the observation times, *t*, indicating the internal dynamics in both the protein and ssDNA are not converged on the MD time window of 100 ns.

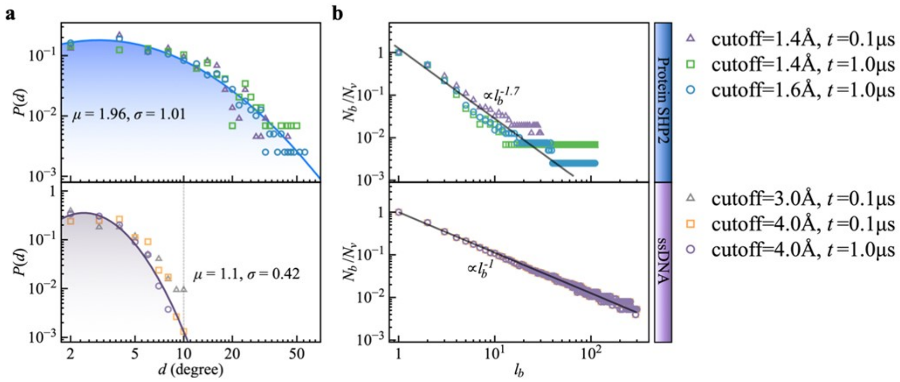

Fig. S11 | Analysis of conformational transition network obtained by using different RMSD cutoff and trajectory lengths (100 ns or 1 μs). (a) The degree distribution $P(d)$ of the protein (top) ssDNA (bottom) transition networks. The blue and purple lines represent log-normal fits (Supplementary Equation 8) for protein and ssDNA, where the μ and σ are fit parameters of the log-normal function. We show the data vanish at the degree of 10 (gray dashed line) for ssDNA, while the distribution of the protein extends to much larger values of d. **(b)** We applied a box covering algorithm[36,37,40] to the CCTN to derive the fractal dimension of three different protein transition networks. The number of boxes (N_b) required to cover the CCTN normalized by the total number of nodes (N_v) in the network is plotted as a function of the box's length, l_b. The power-law fit (blacked) suggests the underlying protein energy landscape is a self-similar fractal with a dimension ~1.7. The number of boxes (N_b) shows a linear relationship with box length (l_b) for single-stranded DNA (ssDNA, bottom panel), indicating the energy landscape of ssDNA is relatively flat with the one-dimensional geometry of CCTN rather than fractal.

Supplementary Tables

Table S1. The sequence of single-stranded DNA (ssDNA, dT_{50}) was studied by smFRET. The single strand of ssDNA is labeled with cy3 and cy5 dye at 50 monomer separations. The letters marked by green and red (underlined) are the corresponding labeling sites on the strand, respectively. The oligonucleotides were purchased from Sangon Biotech Co., Ltd (Shanghai, China)

Sample	Donor/Acceptor	Sequence
single-stranded DNA (ssDNA)	Cy3/Cy5	biotin-5'-CAGCGGACTGCAGTCTCAGCTTTTTTTTTTTTTTTTTTTTTTTTTTTTTTTTTTTTTTTTTTTTTTTTTTT-3'

Supplementary References

1 G. Zhu, J. Xie, W. Kong, J. Xie, Y. Li, L. Du, Q. Zheng, L. Sun, M. Guan, H. Li, T. Zhu, H. He, Z. Liu, X. Xia, C. Kan, Y. Tao, H. C. Shen, D. Li, S. Wang, Y. Yu, Z.-H. Yu, Z.-Y. Zhang, C. Liu and J. Zhu, *Cell*, 2020, **183**, 490-502.e418.

2 Y. Tao, J. Xie, Q. Zhong, Y. Wang, S. Zhang, F. Luo, F. Wen, J. Xie, J. Zhao and X. Sun, *J. Biol. Chem.*, 2021, **296**, 100538.

3 J. W. Chin, S. W. Santoro, A. B. Martin, D. S. King, L. Wang and P. G. Schultz, *J. Am. Chem. Soc.*, 2002, **124**, 9026-9027.

4 J. Xie, X. Si, S. Gu, M. Wang, J. Shen, H. Li, J. Shen, D. Li, Y. Fang and C. Liu, *J. Med. Chem.*, 2017, **60**, 10205-10219.

5 J. A. Lamboy, H. Kim, K. S. Lee, T. Ha and E. A. Komives, *Proc. Natl. Acad. Sci. U. S. A.*, 2011, **108**, 10178-10183.

6 S. Preus, S. L. Noer, L. L. Hildebrandt, D. Gudnason and V. Birkedal, *Nat. Methods*, 2015, **12**, 593-594.

7 R. Roy, S. Hohng and T. Ha, *Nat. Methods*, 2008, **5**, 507-516.

8 S. A. McKinney, C. Joo and T. Ha, *Biophys. J.*, 2006, **91**, 1941-1951.

9 A. D. MacKerell Jr, D. Bashford, M. Bellott, R. L. Dunbrack Jr, J. D. Evanseck, M. J. Field, S. Fischer, J. Gao, H. Guo and S. Ha, *J. Phys. Chem. B*, 1998, **102**, 3586-3616.

10 A. D. Mackerell Jr, M. Feig and C. L. Brooks III, *J. Comput. Chem.*, 2004, **25**, 1400-1415.

11 W. L. Jorgensen, J. Chandrasekhar, J. D. Madura, R. W. Impey and M. L. Klein, *J. Chem. Phys.*, 1983, **79**, 926-935.

12 S. Nosé, *J. Chem. Phys.*, 1984, **81**, 511-519.

13 M. Parrinello and A. Rahman, *J. Appl. Phys.*, 1981, **52**, 7182-7190.

14 T. Darden, D. York and L. Pedersen, *J. Chem. Phys.*, 1993, **98**, 10089-10092.

15 U. Essmann, L. Perera, M. L. Berkowitz, T. Darden, H. Lee and L. G. Pedersen, *J. Chem. Phys.*, 1995, **103**, 8577-8593.

16 B. Hess, *J. Chem. Theory Comput.*, 2008, **4**, 116-122.

17 M. Abraham, D. van der Spoel, E. Lindahl and B. Hess, *Google Scholar There is no corresponding record for this reference*.

18 K. Lindorff-Larsen, S. Piana, K. Palmo, P. Maragakis, J. L. Klepeis, R. O. Dror and D. E. Shaw, *Proteins: Struct., Funct., Bioinf.*, 2010, **78**, 1950-1958.

19 H. J. Berendsen, J. v. Postma, W. F. van Gunsteren, A. DiNola and J. Haak, *J. Chem. Phys.*, 1984, **81**, 3684-3690.

20 R. Metzler, J.-H. Jeon, A. G. Cherstvy and E. Barkai, *Phys. Chem. Chem. Phys.*, 2014, **16**, 24128-24164.

21 Y. Meroz and I. M. Sokolov, *Phys. Rep.*, 2015, **573**, 1-29.

22 J. H. Jeon, E. Barkai and R. Metzler, *J. Chem. Phys.*, 2013, **139**, 121916.

23 J.-H. Jeon and R. Metzler, *Phys. Rev. E*, 2010, **81**, 021103.

24 Y. Mardoukhi, J.-H. Jeon and R. Metzler, *Phys. Chem. Chem. Phys.*, 2015, **17**, 30134-30147.

25 I. Wong, M. Gardel, D. Reichman, E. R. Weeks, M. Valentine, A. Bausch and D. A. Weitz, *Phys. Rev. Lett.*, 2004, **92**, 178101.

26 M. Levin, G. Bel and Y. Roichman, *J. Chem. Phys.*, 2021, **154**, 144901.

27 S. A. Tabei, S. Burov, H. Y. Kim, A. Kuznetsov, T. Huynh, J. Jureller, L. H. Philipson, A. R. Dinner and N. F. Scherer, *Proc. Natl. Acad. Sci. U. S. A.*, 2013, **110**, 4911-4916.

28 J. H. Jeon, V. Tejedor, S. Burov, E. Barkai, C. Selhuber-Unkel, K. Berg-Sorensen, L. Oddershede and R. Metzler, *Phys. Rev. Lett.*, 2011, **106**, 048103.
29 A. V. Weigel, B. Simon, M. M. Tamkun and D. Krapf, *Proc. Natl. Acad. Sci. U. S. A.*, 2011, **108**, 6438-6443.
30 C. Manzo, J. A. Torreno-Pina, P. Massignan, G. J. Lapeyre Jr, M. Lewenstein and M. F. G. Parajo, *Phys. Rev. X*, 2015, **5**, 011021.
31 S. Burov, J. H. Jeon, R. Metzler and E. Barkai, *Phys. Chem. Chem. Phys.*, 2011, **13**, 1800-1812.
32 Y. Golan and E. Sherman, *Nat. Commun.*, 2017, **8**, 15851.
33 A. Weron, K. Burnecki, E. J. Akin, L. Solé, M. Balcerek, M. M. Tamkun and D. Krapf, *Scientific reports*, 2017, **7**, 1-10.
34 A. D. Fernández, P. Charchar, A. G. Cherstvy, R. Metzler and M. W. Finnis, *Phys. Chem. Chem. Phys.*, 2020, **22**, 27955-27965.
35 J. Li, *Phys. Chem. Chem. Phys.*, 2022.
36 X. Hu, L. Hong, M. D. Smith, T. Neusius, X. Cheng and J. C. Smith, *Nat. Phys.*, 2016, **12**, 171.
37 C. Song, S. Havlin and H. A. Makse, *Nature*, 2005, **433**, 392.
38 E. W. Montroll and M. F. Shlesinger, *Proc. Natl. Acad. Sci. U. S. A.*, 1982, **79**, 3380-3383.
39 I. Antoniou, V. V. Ivanov, V. V. Ivanov and P. Zrelov, *Physica D: Nonlinear Phenomena*, 2002, **167**, 72-85.
40 L. K. Gallos, C. Song, S. Havlin and H. A. Makse, *Proc. Natl. Acad. Sci. U. S. A.*, 2007, **104**, 7746-7751.
